# Scale development to measure the patient perception of patient-centered care of dentists in primary care settings of Thailand: a measurement invariance test

**DOI:** 10.1186/s12903-023-03331-1

**Published:** 2023-09-02

**Authors:** Yutthana Khamnil, Surasak Kao-iean, Pagaporn Pantuwadee Pisarnturakit

**Affiliations:** 1 Graduate Program in Dental Public Health, Faculty of Dentistry, Chulalongkorn University, Patumwan, Bangkok, 10330 Thailand; 2 Research Unit of CYD and Department of Educational Research and Psychology, Faculty of Education, Chulalongkorn University, Patumwan, Bangkok, 10330 Thailand; 3 Department of Community Dentistry, Faculty of Dentistry, Chulalongkorn University, 34 Henry Dunant Road, Patumwan, Bangkok, 10330 Thailand

**Keywords:** Confirmatory factor analysis, Exploratory factor analysis, Measurement invariance, Patient-centered dental care, Patient perception, Primary care

## Abstract

**Background:**

Patient-centered care is essential for providing quality services thoroughly at the primary care level, but it is unclear and lacks measurement. This study aimed to develop a reliable and valid instrument to measure patient perception of patient-centered care in primary dental care in Thailand and test the measurement invariance between large and small community hospitals.

**Methods:**

The initial set of 45 items for the patient perception of Patient-Centered Care of Dentist Scale (PCCDS-P version) was developed using a mixed-method approach, which included a literature review, a content validity test, cognitive interviews, and a pre-test. A multistage sampling strategy was used to recruit dental patients or their parents or caregivers from community hospitals across Thailand. Validity was examined through exploratory factor analysis (EFA) and confirmatory factor analysis (CFA). Reliability was assessed using Cronbach's alpha coefficient and the intraclass correlation coefficient. Furthermore, a multi-group analysis was conducted to compare the responses of patients from large and small community hospitals.

**Results:**

Three hundred thirty-six and One thousand one hundred sixty-seven samples were randomized for EFA and CFA, respectively. The final PCCDS-P version consists of 7 factors with satisfactory reliability and validity and is composed of 42 items: dentist-patient relationship, disease-illness, integrated care, communication, shared information and decision-making, holistic, and empathy and anxiety management. The CFA showed the model fit was consistent with the entire sample. The metric invariance analysis showed that the factor loadings were invariant across patient groups. Overall, Cronbach's alpha coefficient and intraclass correlation coefficient were satisfactory.

**Conclusions:**

The newly developed PCCDS-P version is composed of seven domains with 42 items with good reliability and validity, and it indicated measurement invariance across patients in large and small community hospitals.

## Introduction

Thailand's healthcare system has prioritized delivering high-quality services since the implementation of universal health coverage in 2002 [1]. In 2019, the Primary Care Act B.E. 2562 was introduced to ensure accessible, equitable, and cost-effective primary healthcare services [2]. Patient-centered care has been identified as a crucial element in ensuring quality care, as studies have shown its potential to improve health outcomes. Some extents related to patient-centered care were studied, and it was found that enhancing patient participation can have a good effect on health-related quality of life, according to a Hong Kong study that looked at how it relates to that quality of life in primary care [3]. Communication that is clear and effective can improve patient happiness, disease understanding, drug compliance, and ultimately healthcare results. When patients actively participate in their dental care decisions, they are more likely to understand their conditions and treatment options. This understanding can lead to better compliance with treatment plans and improved treatment outcomes. Patients who feel involved and valued in their care are also more likely to maintain long-term relationships with their dentists, leading to continuity of care and better overall oral health [4, 5].

All primary care professionals, including those in the dental field, must focus on patient-centered care and clinical excellence. Since 1994, the Thai dental care system has been integrated into primary care, with dentists and dental nurses serving as the primary providers in community (or district) hospitals and sub-district health centers [6]. Almost all primary care dentists work in community hospitals, which have four sizes depending on the number of inpatient beds. Most of their work is close to the function of integrated primary care [7]. Although there are many private dental clinics, they frequently concentrate on secondary and tertiary services.

Although patient-centered care in dentistry is essential, its definition and interpretation are unclear, especially in primary care [8–10]. Even more difficult is the measurement development for properly incorporating all concepts aiming for better oral health. Many instruments are used for measuring some extents that are relevant to patient-centered care in dental care, but most of them focus on satisfaction, communication, and dental office management [11–17]. There is a rare instrument for measuring patient-centered care that focuses on the interpersonal action between dentist and patient in primary care [18, 19]. Hence, there is a need for a reliable and valid instrument to evaluate patient-centered care in primary care dentistry.

Based on studies in Indonesia in dental care settings, it was found that different types of dental care settings can affect the level of responsiveness and empathy of patients, which is similar to the patient-centered care concept [20, 21]. Some studies in Thailand show that patient perceptions of responsiveness differ depending on the type of hospital [22, 23]. Therefore, to create a trustworthy instrument, it is necessary to assess the measurement invariance of the PCCDS-P version between various sizes in large and small community hospitals in Thailand. This new tool should be able to accurately gauge how patients view the patient-centered care that dentists provide. It can assist decision-makers in better understanding the efficacy of patient-centered care strategies, identifying areas for development, and allocating resources.

## Methods

### Population and sample

The study obtained the ethical approval of the Human Research Ethics Committee of the Faculty of Dentistry, Chulalongkorn University, Bangkok, Thailand (study code HREC-DCU 2021–113). The samples were patients or parents or caregivers, ages 18–70, in the waiting area of the dental department of a community hospital in Thailand who had had at least two dental visits within the past 12 months. A good sample size for EFA was recommended by Costello and Osborne in 2005 [24] at least 300 samples. We needed a large value of the parameters for determining sample size for the invariance test, such as the degree of freedom, which we set at 24, because our pre-specified patient-centered care components are twelve, following our prior work [25]. The sample size was calculated online at the Computing Power and Minimum Sample Size for RMSEA website [26] with alpha = 0.05, power = 0.95, degree of freedom = 24, a null hypothesis with RMSEA = 0.06, and an alternative hypothesis with RMSEA = 0.02. The sample size was 482, and we added 10% of incomplete data, so the sample size was at least 532 patients for each group of small (30 in-patient department (IPD) beds) and large (31–60 IPD beds) community hospitals, so a total of 1,364 calculated samples were needed. The multi-stage random sampling was applied, starting with simple random selection for 5–7 provinces of the four regions of Thailand, followed by purposive sampling for 1–2 small or large community hospitals in each province. The convenient sampling method was applied to get 40–60 patients from each hospital.

### Initial item generation and pre-testing

We used our previous qualitative study to analyze patient-centered dentistry through in-depth interviews with 5 experts, 7 dental practitioners, and 8 patients and a review of existing instruments used to measure patient-centered care or similar extents in dental and related medical and nursing care [25]. It found twelve components of patient-centered care provided by dentists in primary care, which include communication, holistic care, empathy, disease and illness management, shared information and decision-making, the dentist-patient relationship, continuity of care, coordination, accessibility, anxiety management, and the self-awareness of the dentist. The 61 initial items were generated from this study. We modified some of the items from literature reviews that contain ideas comparable to our twelve components, such as those from Naorungroj S. et al. (2018) for items ID cp1.35, co2.30, an3.28, sd5.15, and sd3.13, and those from Hojat et al. (2009) for items ID em1.23 of this present study (as seen in Table 2).

Content validity was evaluated by 5 experts using the content validity index, which was 94.75% excellent validity [27]; 2 items were dropped. The cognitive interviewing with five patients was conducted to refine and assess item interpretation and finalize item structure. A pre-test was conducted with 97 patients in two community hospitals in Phra Nakhon Si Ayutthaya and Nakhon Pathom province to assess the reliability of the questionnaire. 14 items were deleted due to a low corrected item-total correlation between 0.008 and 0.345. The Cronbach’s alpha coefficient of the preliminary 45-item scale was 0.95, which indicated high reliability [28].

### Data collection

The paper-based, self-administered questionnaires were distributed to the dental departments of 32 large and small community hospitals in 26 provinces across Thailand by parcel services. Dental patients or their parents or caregivers, who had received the dental treatment, were informed and asked for consent to participate in the study from July to October 2022. We monitored the response every 3–4 weeks. If the response rate was relatively low, we followed up every two weeks to increase participation. Before returning the questionnaires to the researchers by parcel services, dental staff reviewed them for any missing information.

### Data analysis

All data analyses were conducted using IBM SPSS Statistics software version 24 and IBM SPSS Amos version 22. After data collection, thoroughly clean and organize the dataset and check for data entry errors and inconsistencies. A complete case analysis was applied if the missing data was less than 5%. The collected data were randomized for exploratory factor analysis (EFA) and confirmatory factor analysis (CFA). We used descriptive statistics and the chi-square test of independence to compare how the demographic characteristics of the split samples were spread out, especially for hospital size, to see if there was a significant link between the variables and the groups. Further statistics were conducted to assure that the EFA, CFA, and measurement invariance assumptions were met.

The EFA was employed to capture the underlying factor. The items with factor loading greater than 0.4 were retained on a single factor. Items with similar loadings on more than one factor were deleted [24]. The CFA was employed to confirm the model's fit with the evidence data. Fit statistic criteria included comparative fit index (CFI; criterion > 0.95), Tucker-Lewis index (TLI; criterion > 0.95), root mean square error of approximation (RMSEA; criterion 0.06), and Akaike information criterion (AIC) [29, 30]. Internal consistency and construct reliability (CR) greater than 0.7 indicate good reliability, and average variance extracted (AVE) greater than 0.5 indicate good convergent validity [31].

The multilevel measurement invariances between patients in large and small community hospitals were looked at by comparing the baseline measurement model (configural invariance) and the upper level of the invariance test. It examines if the constructions' distribution of free and fixed loadings is the same across various groups. Testing for metric invariance comes next, and the equivalent loadings on the factors are evaluated. For scalar invariance, investigate whether item intercepts are equivalent. It makes sure that all the mean differences in the shared variance of the items are captured by the mean differences in the latent construct. Testing for residual invariance is the last step in proving measurement invariance. This stage evaluates if item residuals for metric and scalar invariant items are equivalent. Invariance is not supported if the overall model fit is significantly inferior (*p*-value less than 0.001 and difference in CFI equal to or less than 0.01) [32, 33].

The intraclass correlation coefficient was analyzed for evaluating the test–retest reliability of the new 42-item scale by distributing the questionnaire to 30 patients at Phrasamutjedee community hospital twice (one week apart) [34].

## Results

### Characteristics of the participants

One thousand five hundred twenty-seven patients responded to the questionnaire, or 47 responses per hospital on average. 24 incomplete responses were dropped. 336 and 1167 participants were randomized into EFA and CFA, respectively. Most participants (68.9%) were female, and their mean age was 41.05 ± 16.05 years. The majority of them (48.7%) have government universal coverage scheme insurance. The demographic distribution of the split sample data was displayed in Table 1.

**Table 1:** Characteristics of the EFA and CFA sample, Total *N *= 1503

**Characteristics**	**n (%)**		
	**EFA**	**CFA**	*p*-value*
	**(*n *= 336)**	**(*n *= 1167)**	
**Gender**			
Male	110 (32.7)	358 (30.7)	0.47
Female	226 (67.3)	809 (69.3)	
**Age (Yrs.)** Mean 41.05 (16.05)			
18–29	97 (28.9)	340 (29.1)	0.63
30–39	64 (19.0)	234 (20.1)	
40–49	62 (18.5)	198 (17.0)	
50–59	58 (17.3)	233 (20.0)	
60–70	55 (16.4)	162 (13.9)	
**Highest education**			
Primary school	57 (17.0)	237 (20.3)	0.40
High school	144 (42.9)	482 (41.3)	
Bachelor and higher	135 (40.2)	448 (38.4)	
Insurance**			
CSMBS	98 (29.2)	354 (30.3)	0.56
UCS	172 (51.2)	560 (48.0)	
SSS	66 (19.6)	253 (21.7)	
**Hospital size**			
Small (≤ 30 beds)	191 (51.2)	631 (54.1)	0.35
Large (> 30 beds)	145 (48.8)	536 (45.9)	
**Frequency dental visit within 24-month**			
2 times	152 (45.2)	587 (50.3)	0.26
3 times	123 (36.6)	383 (32.8)	
> 3 times	61 (18.2)	197 (16.9)	

* Pearson χ2 test *P*-value, ** *CSMBS * Civil Servant Medical Benefit Scheme; UCS = Universal Coverage Scheme, SSS = Social Security Scheme

The chi-square tests indicated that the two groups were similar in all characteristics. As a result of the item analysis, the mean score of each of the 45 items was 3.62–4.34, and the range of the standard deviation was 0.81–1.15. All items have skewness and kurtosis within a normal range of ± 2.0 [35] (*data not shown*).

## Exploratory factor analysis

EFA results for evaluating construct validity revealed factorability: the Kaiser–Meyer–Olkin (KMO) = 0.958, and Bartlett's test of sphericity *p*-value < 0.001 (χ^2^ = 16,521.43). A series of EFA models ranging from five to seven factors based on parallel analysis were compared. Three items were considered to be cross-loaded on two factors with similar loading, and the difference in factor loading value was less than 0.20 [36]. The most-fitting seven-factor model using varimax rotation with parallel analysis contained 42 items. The four prespecified factors were grouped as accessible, continuous, coordinated, and comprehensive into integrated care (IC) as they related to the integrated function of primary care [7]. The two items of anxiety management and the three items of empathy were grouped into empathy and anxiety management (EAM). Two items of the self-awareness component were loaded into the dentist-patient relationship (DP). The fitted model accounted for 76.18% of the total variance explained with 42 items in seven factors: Integrated Care (IC): 11 items; Holistic (HO): 5 items; Communication (CO): 6 items; Dentist-Patient Relationship (DP): 6 items; Empathy and Anxiety Management (EAM): 5 items; Shared Information and Decision-Making (SD): 5 items; Disease and Illness (DI): 4 items. The eigenvalue, factor loading, percentage of the variance, cumulative percentage of variance, and proposed names of the factors are presented in Table 2.

**Table 2:** Factor loading, % of variance and Cumulative % of variance of the PCCDS-P version

Item Id	Item	h^2^	Factor								
	**Description**		**IC**	**HO**	**CO**	**DP**	**EAM**	**SD**		**DI**	
ac2.44	You have received all the treatments you need on this visit	0.789	0.775								
cd1.38	Coordination between dentists and dentists/dental staffs are smooth, comfortable and fast	0.787	0.762								
cn3.42	Your dentist advice for regular oral health check-up	0.738	0.736								
ac1.43	You have received the treatment on time	0.655	0.714								
cn1.40	Your dentist made an appointment for ongoing treatment or follow-up treatment as necessary	0.748	0.697								
cd2.39	Dentist coordinates with other departments such as medical department, dispensing department, cashier based on patient benefits	0.672	0.695								
ac3.45	You can easily access or meet your dentist when you need	0.635	0.682								
cp1.35	The oral examination of your dentist covers all parts of the mouth, not only the teeth or gums where you have problems	0.656	0.614								
cp3.37	9According to your dentist’s advice, you can regularly brush your teeth with fluoride toothpaste twice a day and before bedtime, you reduce consumption of sugary foods, soft drinks, etc	0.686	0.609								
cp2.36	Because of your dentist's explanation, you understand that general health and oral health are related	0.706	0.578								
cn2.41	If there is a change of dentist or a referral to another dentist, the new dentist can continue the treatment smoothly	0.557	0.575								
wp4.4	Your dentist asks about limitations or obstacles related to your care plan such as limited time and travel expenses	0.895		0.894							
wp3.3	Your dentist responds to your oral health opinions	0.873		0.882							
wp5.5	Your dentist considered your limitations or obstacles into your care plan	0.863		0.874							
wp1.1	Your dentist asks about your general life, such as where your house is, work, study, family, and friends and including belief and spirituality	0.717		0.807							
wp2.2	Your dentist considered your general life and included belief and spirituality into your care plan	0.698		0.762							
co5.33	Your dentist uses equipment or media such as brochures, pictures, videos, or x-ray films his/her explanations and advice	0.748			0.766						
co4.32	Your dentist talks with easy words, not too many medical terms	0.781			0.750						
co1.29	Your dentist has a friendly greeting	0.772			0.722						
co6.34	Your dentist asks you to ask if you had any doubts, questions, or did not understand any point	0.725			0.696						
co2.30	Your dentist gave you enough time to describe your symptoms and illness	0.678			0.636						
co3.31	Your dentist attentively listens, makes eye contact, and looks at you more than looking at documents or computer screens	0.562			0.469						
dp3.18	I trust my dentist	0.801				0.713					
dp4.19	I am confident that I will be able to properly maintain my own oral health when I am taken care of by the dentist here	0.832				0.697					
dp2.17	I have never had any worries when my oral health was taken care of by the dentist here	0.737				0.641					
sa2.22	Your dentist can manage their emotions well even in difficult situations involving caring for you	0.723				0.632					
sa1.21	Your dentist is in a normal mood and enthusiastic while serving you	0.740				0.610					
dp5.20	Your dentist takes very good care of you and made you come back here regularly	0.784				0.608					
em3.25	After the dental treatment, your dentist asks or gives you an opportunity to share your feelings and opinions about that treatment	0.881					0.759				
em2.24	Your dentist clearly shows sympathy despite the complexity and difficulty of your treatment procedure	0.876					0.741				
em1.23	Your dentist understands and sees illness or worries in the same as your perspective	0.850					0.700				
an3.28	Your dentist asks periodically about your pain while providing dental treatment	0.800					0.680				
an2.27	Your dentist reminds you before doing some procedures that may cause pain, such as starting to inject an anesthetic, starting to grind the teeth	0.740					0.590				
sd5.15	Your dentist explains advantages, disadvantages, costs, treatment options, and possible treatment outcomes	0.894						0.757			
sd3.13	Your dentist gives you (and your relatives) the opportunities to join in setting treatment goals	0.847						0.733			
sd2.12	Your dentist enhances you (and your relatives) to participate in decision-making to choose the appropriate treatment for you	0.827						0.693			
sd4.14	Your dentist describes the treatment plan and procedures, as well as the duration of the treatment	0.810						0.687			
sd1.11	Because of Your dentist's explanation, you know that the treatment outcome depends on the cooperation between you and your dentist	0.701						0.573			
di2.7	Your dentist asks about your idea of what you are sick with	0.834								0.723	
di5.10	Your dentist asks about your expectation of this visit	0.838								0.716	
di3.8	Your dentist lets you tell your worries about your oral health	0.824								0.679	
di4.9	Your dentist asks about the impact of oral illness on your daily life, work, or school	0.714								0.603	
Eigenvalue			8.063	5.074	4.453	3.952	3.862		3.747		2.842
% of Variance			19.198	12.082	10.602	9.409	9.194		8.921		6.767
Cumulative % of variance			19.198	31.280	41.883	51.292	60.487		69.407		76.175

*IC* Integrated Care, *HO* Holistic, *CO*  Communication, *DP*  Dentist- Patient relationship, *EAM * Empathy and Anxiety management, *SD*  Shared information and Decision-making, *DI*  Disease and Illness, *sa*  Dentist’s self-awareness, *cp* Comprehensive care, *cn* Continuous care, *cd*  Coordinated care, wp Whole person (changed to HO = holistic in the final analysis in CFA), h^2^ = Communality

### Confirmatory factor analysis, invariance test and reliability test

**Fig. 1:**
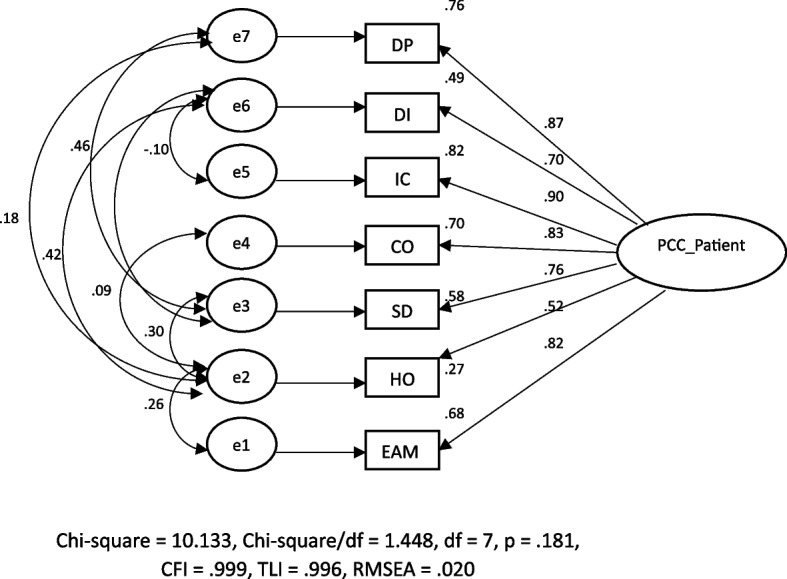
Patient perception of the patient-centered care of dentist measurement model IC = Integrated Care, HO = Holistic, CO = Communication, DP = Dentist-Patient relationship, EAM = Empathy and Anxiety management, SD = Shared information and decision-making, DI = Disease and Illness

The overall model fit of the initial measurement model of the PCCDS-P version was evaluated. The results revealed inadequate model fit with the data, as indicated by χ^2^ = 463.460, χ^2^ /df = 33.104, df = 14, *p* < 0.001, CFI = 0.925, TLI = 0.888, RMSEA = 0.166, and AIC = 491.461. A series of modifications were made in an effort to achieve parsimony using the Akaike information criteria, where a lower value denotes a better match. The final CFA revealed the fit indices of the model as follows: χ^2^ = 10.113, χ^2^/df = 1.448, df = 7, *p* = 0.181, CFI = 0.999, TLI = 0.998, RMSEA = 0.020, and AIC = 52.133 (Fig. 1). The factor loadings were significant and ranged from 0.52 to 0.87. The CR was 0.91 and the AVE was 0.61 (Tables 3 and 4). The series of multilevel measurement invariance tests started with individual split data from large and small community hospitals. Testing for the large hospital demonstrated χ^2^ = 8.996, χ^2^/df = 1, df = 9, *p* = 0.438, CFI = 1.000, RMSEA = 0.012, the small hospital demonstrated χ^2^ = 4.975, χ^2^/df = 0.995, df = 9, *p* = 0.419, CFI = 1.000, RMSEA = 0.009, and the configural model showed χ^2^ = 7.952, χ^2^/df = 0.994, df = 8, *p* = 0.438, CFI = 1.000, RMSEA = 0.000. The addition of constraints for equal factor loadings still showed an adequate fit, which indicated metric invariance. The incremental addition of constraints on variable intercepts and on all estimated error terms did not fit satisfactorily on all fit indices, providing no evidence for scalar and error invariances. All models and fit indices are shown in Table 4.

**Table 3:** Mean, SD, Factor Loading, R^2^, Cronbach’s alpha, AVE, and CR of the PCCDS-P version measurement model

Component	Mean^a^	SD	Loading	R^2^	Cronbach’s alpha
1. Dentist-patient relationship: DP	6.60	1.15	0.87	0.76	0.93
2. Disease and illness: DI	5.85	1.29	0.70	0.49	0.90
3. Integrated care: IC	7.01	1.22	0.90	0.81	0.95
4. Communication: CO	5.63	1.11	0.83	0.70	0.92
5. Share information and decision-making: SD	6.39	1.26	0.76	0.58	0.91
6. Holistic: HO	4.59	1.17	0.52	0.27	0.92
7. Empathy and anxiety management: EAM	6.39	1.24	0.82	0.67	0.93
Construct reliability (CR)					0.91
Average variance extracted (AVE)					0.61

^a^The mean is calculated from the average of the sum of the factor scores of items in each component

**Table 4:** CFA models and invariance tests

Model	χ^2^	df	*P*-value	χ^2^/df	RMSEA	CFI	AIC
Model_0 (initial)	463.460	14	< 0.001	33.104	0.166	0.925	491.461
Model_1	266.050	13	< 0.001	20.465	0.129	0.958	296.050
Model_2	241.205	12	< 0.001	20.100	0.128	0.962	273.255
Model_3	125.044	11	< 0.001	11.368	0.094	0.981	159.044
Model_4	57.222	10	0.002	5.722	0.064	0.992	93.222
Model_5	25.827	9	0.002	2.870	0.040	0.997	63.827
Model_6	14.812	8	0.027	1.863	0.027	0.999	54.312
Model_7(final) **Invariant test**	10.133	7	0.181	1.448	0.020	0.999	52.133^a^
Model_large hospital	8.996	9	0.438	1.000	0.012	1.000	
Model_small hospital	4.973	5	0.419	0.995	0.009	1.000	
Model_A Configural invariance	7.952	8	0.438	0.994	0.000	1.000	
Model_B Metric invariance	16.40	14	0.290	1.172	0.012	1.000	
Model_C Scalar invariance	48.71	15	< 0.001**	3.247	0.044	0.994	
Model_D Residual invariance	113.8	32	< 0.001**	3.555	0.047	0.986	
Comparison test *							
Model B-A	8.449	6	0.207			< 0.001	
Model C-B	32.307	1	< 0.001**			0.005***	
Model D-C	65.062	17	< 0.001**			0.008***	

- Model_0 – Model_7 analyzed on 1167 samples, Model-large analyzed on 552 samples of large hospital subgroup, Model_small analyzed on 615 samples of small hospital subgroup

- Model_1, covariance between errors SD and DI, Model_2, covariance between errors HO and CO, Model_3, covariance between errors HO and DI, Model_4, covariance between errors SD and HO, Model_5, covariance on between errors SD and DP, Model_6, covariance between errors IC and DI, Model_7, covariance between errors HO and EAM (as seen in Fig. 1)

*Abbreviations*: *AIC* Akaike's information criterion, *CFI* Comparative fit index, *df* Degree of freedom, *GFI* Goodness-of-fit index, *RMSEA* Root mean square error of approximation, *IC*  Integrated Care, *HO*  Holistic, *CO*  Communication, *DP* Dentist- Patient relationship, *EAM*  Empathy and Anxiety management, *SD*  Shared information and Decision-making, *DI* Disease and Illness

^a^lowest AIC

^*^Difference of χ2, df, and CFI, ** *p*-value < 0.001 means invariance not supported, ***⍙CFI ≤ 0.01 means invariance supported

The range of Cronbach’s alpha coefficient for each component was 0.90–0.95, and overall, it was 0.93. The intraclass correlation coefficient of this new 42-item scale was 0.90 *(data not shown).*

## Discussions

The final EFA revealed a seven-factor model that adequately explains the variation in 42 items. The other five pre-specified components were dropped. However, some items that ever belonged to dropped components were loaded onto other components, then moved to those components. For instance, item ID sa1.21–sa2.22 (as seen in Table 2) had previously belonged to the component "Dentist's self-awareness," but following EFA, they firmly loaded onto the component "Dentist-patient interaction" and switched to it. This indicated a less complicated interpretation of patients than that of the literature and experts.

The dentist-patient relationship (DP) entails establishing a positive connection between the dentist and their patient, where mutual respect is demonstrated by the dentist towards the patient, leading to trust and confidence from the patient towards their dentist. This type of relationship fosters better dental care and encourages patients to take responsibility for their own oral health. This dentist-patient relationship is in harmony with the long-term relationship between dentists described by Scambler S. et al. [37] and the building of mutual trust described by Noushi N. et al. [38]. But in the study of Rozier RG. et al. 2019 [18], to construct a scale to measure the patient-centered dental home, they found no similarities to this present study.

The term “Disease-Illness” (DI) pertains to the dentist's skill in comprehensively examining the patient's illness experience, encompassing their understanding of the disease, their emotions towards the illness, how it affects their daily functioning, and the patient's anticipated treatment and outcome expectations. These come into common with the studies of Kulich et al., 2003, and Apelian et al., 2014 [39, 40], which highlighted that dentists need to understand both the experience of patient illness and disease. However, this is different from Rozier GR. et al., 2019 [18], and Naoungroj S. et al., 2018 [41], who do not include this component in their instrument.

Integrated care (IC) encompasses four crucial aspects of primary care, namely accessibility, continuity, coordination, and comprehensive care. It emphasizes the dentist's ability to address these issues independently without relying on the dental office administration. This component is in accordance with the patient-centered dental home model of Damiano et al., 2015–2019 which was highlighted in primary care [19, 42], and was similar to Rozier RG. et al. (2019)(18) and Naoungroj S. et al. (2018) [41].

The communication (CO) aspect pertains to the dentist's capacity to effectively communicate with patients and their family members. It involves using simple and understandable language, actively listening, allowing sufficient time for communication, and utilizing various media for clear explanations, all of which are essential components identified in Hunsrisakun's 2010 study [43], Mills et al.'s 2015 research [44], Rozier RG. et al., 2019 [18] and Naoungroj S. et al., 2018 [40],and Loignon et al.’s 2010 analysis [45].

Empathy and anxiety management (EAM) encompass two vital aspects of dental care, namely compassion and anxiety management. Dentists must strive to empathize with their patients, seeing the illness from their perspective, a trait commonly identified in various models [38, 43, 44]. Additionally, they must possess the competency to provide gentle procedures while considering the patient's pain sensation and effectively managing their dental anxiety or fear, particularly with patients suffering from dental phobia [39, 46]. However, this cannot be found in the final result of Rozier RG. et al.’s 2019 study [18].

The shared information and decision-making (SD) and holistic (HO) components are the most frequently cited components in the literature. This could be attributed to the fact that dental treatment often offers multiple options for a given oral health condition [19, 37–40, 43, 44], making shared information and decision-making critical in promoting patient involvement in their dental care. Furthermore, dentists should acknowledge the impact of the patient's family context, residential communities, personal beliefs and spirituality, education, religion, ethnicity, occupation, lifestyle, and environment, which are factors that can influence the patient's treatment plan. This personalized approach to care is a common theme across the literature reviewed [18, 19, 38–41, 43–45, 47].

All CFA model fit indices indicated the structure of the PCCDS-P version was consistent with the empirical data (χ2 = 10.113, χ2/df = 1.448, df = 7, p = 0.181, CFI = 0.999, TLI = 0.998, RMSEA = 0.020, AIC = 52.133). Furthermore, the convergent validity test shows that the constructs constituting the scale are correlated with each other (AVE = 0.61). The seven factors together confirm that they can measure the patient's perception of the patient-centered care provided by dentists in primary care. Three components of this study, integrated care (IC), dentist-patient relationship (DP), and communication (CO), are in harmony with 10 items of three constructs (accessible and comprehensive care, compassionate care, and health-literate care) from the study of Rozier.RG. et al., 2019 [18]. In addition, our results revealed other four factors: empathy and anxiety management (EAM), holistic (HO), disease and illness (DI), shared information, and decision-making (SD). It's possible that Thai culture, which is influenced by Buddhism and values sincerity and respect, has had an impact on this. A highly regarded quality in Thai society is empathy, in particular [48]. Thai people always ask permission before acting in a way that can cause harm to others, or they express their regret either verbally or nonverbally.

The measurement invariance test between patients in different sizes of hospitals (large community hospitals and small community hospitals) indicated metric or weak invariance, which means the structure of the factors and factor loadings are invariant. This may be due to the fact that patients in larger hospitals may have more specialized dental procedures that require more visits and time than those in smaller hospitals. The dental office management and patient waiting list might be different. However, this instrument can be used for measuring patient perceptions of patient-centered care by dentists in primary care settings by separately interpreting between small and large community hospitals.

There are several studies of instruments to measure dental care satisfaction and quality from a patient perspective that have some questions in common with our study [11, 14, 15, 17, 18, 41, 49]. However, most of them did not reveal their validity or invariance. As seen in Table 5, there are some similarities and variations between this study and the two relevant instruments.

**Table 5:** Comparison of the two most relevant studies to this present study

	PCCDS-P version	PCDHS	PFPCDC
Authors	This present study	Rozier RG., et al. in 2019 [18]	Naorungroj S., et al. in 2018 [41]
Samples	1,503 patients in large and small community hospitals, Thailand	893 mothers, native English and Spanish speaking, USA	412 patients in a dental school, Thailand
Construct validity	EFA, CFA, and Invariance test	EFA, CFA, and Invariance test	Not reported
Components/ items	7/42	3/10	4 steps of dental procedure/21
Similarities and differences of components and items	1. Dentist-patient relationship: DP	No similar	Step 4 Oral health promotion (only 1 item similar)
	2. Disease and illness: DI	No similar	No similar
	3. Integrated care: IC	Accessible and comprehensive care	Step 4 Oral health promotion
	4. Communication: CO	Compassionate care,	Step 1 Data collection
		Health literate care	
	5. Share information and decision-making: SD	No similar	Step 2 Plan
	6. Holistic: HO	Compassionate care	Step 1 Data collection
	7. Empathy and anxiety management: EAM	No similar	Step 3 Treatment

*PCCDS-P version*  Patient perception of patient-centered care of dentist scale, *PCDHS* Patient-centered dental home scale, *PFPCD* Patient feedback to patient-centered dental care provided by dental students

The present study aimed to provide a conceptual framework and tool for measuring patient perceptions of patient-centered dental care in primary care settings. The participants were recruited from 32 hospitals in 26 provinces across 4 regions of Thailand; therefore, they do represent patients in primary care in Thailand. The scale can be used as a reliable tool to assess the patient-centered care of primary care dentists because its reliability and validity are satisfactory.

The current study has identified certain limitations and provided suggestions for future research. While the scale used in the study demonstrated reliability and validity, it requires more than 10 min to complete the 42-item scale. Hence, it would be advantageous to develop a shorter version of the inventory for broader usage. Additionally, reassessing the psychometric properties of the scale in different samples, such as private clinics and higher care levels, would enhance its value.

### Implications

1. All dentists, especially those who provide primary care, should embrace a patient-centered care philosophy since it can improve oral health outcomes and patient satisfaction.
2. The scale's results can be used as one of the performance indicators and also as a guideline for primary dental care administrations to create initiatives focused on providing patient-centered care.
3. Primary care dentists should receive further training in patient-centered care concept as much as they can, whether it be through short refresher courses or longer programs like family dentistry, advanced general dentistry, or primary care dentistry.
4. It is highly recommended that patient-centered care concept be included in all undergraduate dental courses, particularly in clinical training.

## Conclusion

The self-administered questionnaire's robust validity and reliability, encompassing seven distinct components and 42 items, position it as a valuable instrument for appraising patient-centered dental care in primary care settings. Our study has shown metric invariance across patients in both large and small community hospitals, affirming the applicability of the scale across different healthcare contexts. Future research could expand its scope by targeting a wider and more diverse patient population and exploring different geographic regions to enhance the scale's applicability and generalizability. Additionally, the development of a concise version of the inventory could offer a more versatile tool for widespread utilization.

## Data Availability

The datasets generated and/or analyzed during the current study are available from the corresponding author upon reasonable request.

## Abbreviations

AIC: Akaike information criterion
AVE: Average Variance Extracted
CFA: Confirmatory Factor Analysis
CFI: Comparative Fit Index
CR: Construct Reliability
EFA: Exploratory Factor Analysis
IPD: In-patient Department
PCCDS-P version: Patient Perception of Patient-Centered Care of Dentist Scale
RMSEA: Root Mean Square Error of Approximation
TLI: Tucker-Lewis Index
